# Comparison of Race Time-Differences Between and Within Para and Able-Bodied Cross-Country Skiers

**DOI:** 10.3389/fspor.2021.823014

**Published:** 2022-02-08

**Authors:** Camilla H. Carlsen, Cecilia Severin, Øyvind Sandbakk, Julia K. Baumgart

**Affiliations:** Department of Neuromedicine and Movement Science, Centre for Elite Sports Research, Norwegian University of Science and Technology, Trondheim, Norway

**Keywords:** competition, time-factor, classification, performance, Paralympic, nordic skiing

## Abstract

**Purpose:**

To compare differences in race time (i.e., the average percent difference in race time for each skier compared to the winner, RT_diffs_) between female and male Para and able-bodied (AB) skiers, and to examine whether RT_diffs_ change across seasons.

**Methods:**

Race data from World Cups (WCs), World Championships (WCHs), and Paralympic/Olympic Winter Games (PWG/OWGs) of the 2011–2020 seasons was extracted from the website of the International Paralympic Committee and the International Ski Federation. All individual distance races for female and male visually impaired standing (VI), physically impaired sitting (SIT) and standing (STAND), and AB skiers with ≥10 competitors were included in the analyses. We investigated the main effect of skiing groups (i.e., VI, STAND, SIT, and AB skiers) and sex on RT_diffs_ for top-3 and top-8 skiers. Furthermore, the main effect of season and skiing group on RT_diffs_ for top-3 and top-8 skiers were investigated. All models were adjusted for distance, skiing style (classical- and freestyle), and event type (WC, WCH, and PWG/OWG).

**Results:**

RT_diffs_ were significantly larger in Para compared to AB skiers (top-3: 2.1 vs. 0.9%; top-8: 6.2 vs. 2.1%, all *p* < 0.01), and in female compared to male skiers (top-3: 1.8 vs. 1.3%; top-8: 4.9 vs. 3.5%, all *p* < 0.05). For top-3 skiers, RT_diffs_ did not significantly differ between the Para categories (all *p* > 0.2), while for top-8 skiers RT_diffs_ were significantly larger for VI compared to SIT and STAND (7.0 vs. 5.5 and 5.6%, respectively, all *p* < 0.05). RT_diffs_ were stable across the 2011–2020 seasons for top-3 skiers (VI: 1.7–3.6%, STAND: 1.1–2.2%, SIT: 1.0–3.9%, AB: 0.4–1.1%; all *p* > 0.1) and top-8 skiers (VI: 3.4–12.0%, STAND: 2.6–5.7%, SIT: 1.9–5.9%, AB: 0.1–1.7%; all *p* > 0.1).

**Conclusion:**

The larger RT_diffs_ in Para compared to AB skiers indicate larger variability in performance, which are in part disability related. Female skiers displayed larger RT_diffs_ than their male counterparts, indicating larger variability in performance among the female skiers. Our results provide insights about performance demands in Para cross-country skiing, which is of relevance for coaches and skiers.

## Introduction

Understanding how race times differ between competing athletes is crucial for elite athletes and their coaches since they indicate the performance improvements required for winning. This insight can guide athletes in their goal setting and help coaches evaluate the training progress. Numerous studies have investigated differences in race time for able-bodied (AB) endurance athletes in a range of summer (Pyne et al., 2004; Paton and Hopkins, 2005, 2006; Nibali et al., 2011; Smith and Hopkins, 2011) and winter sports (Bullock et al., 2009; Spencer et al., 2014; Skattebo and Losnegard, 2018). However, studies on differences in race times between athletes with a disability (Para) and AB are currently limited to swimming and show larger differences among Para swimmers (Fulton et al., 2009). Even though Para cross-country (XC) skiing is one of the most popular winter Para-sports, research on differences in race time has so far only been conducted on AB skiers (Spencer et al., 2014). This research showed that differences in race time were slightly larger among top-10 female compared to male AB skiers (0.8 vs. 0.1%, respectively) (Spencer et al., 2014).

Both Para and AB skiers compete in annual World Cups (WCs), biennial World Championships (WCHs), and quadrennial Paralympic (PWGs)/Olympic Winter Games (OWGs) (IPC, 2021a). Race courses consist of undulating terrain over distances ranging from 0.8 to 20 km for Para, and 1.2 to 50 km for AB skiers. To improve competition fairness for Para skiers with different disabilities, they are classified into three different categories: (1) physically impaired sitting (SIT) skiers, (2) physically impaired standing (STAND) skiers, and (3) visually impaired standing (VI) skiers (Tweedy and Vanlandewijck, 2011; IPC, 2021a). While STAND and VI skiers compete in both the classical style and free-style, SIT skiers only compete in the classical style (IPC, 2020, 2021a).

Within the three categories, Para skiers are further divided into classes based on the functional impact the disability has on performance: LW10-12 for SIT, LW2-9 for STAND, and B1-3 for VI (IPC, 2021a). Due to often low numbers of competitors in each class, all classes in the same category compete together in a single race. Based on their class, each skier is assigned a time-factor (Vanlandewijck and Thompson, 2011; IPC, 2016; Rosso and Gastaldi, 2020), which is multiplied with the skier's actual race time to determine their adjusted race time and their final rank (Vanlandewijck and Thompson, 2011; IPC, 2016). The purpose of the time-factor is to further reduce disability-related differences in race time between skiers, so that skiers with larger functional limitations receive larger deductions in race time. While it is reasonable to assume that the differences between the adjusted race times would be smaller than the unadjusted ones, this has not yet been investigated in Para XC skiing.

In elite AB XC skiing, the average skiing speed has increased by ~10% over the last three decades for middle- and long-distance classical and free-style races (Losnegard, 2019). Since the differences in race time have remained stable within this time period (Stöggl et al., 2009; Spencer et al., 2014), it seems that the speed increased equally for all skiers. In contrast, smaller race times differences have been reported for Para sprint runners across the 1992–2012 seasons (Grobler et al., 2015), likely due to the growing popularity of Para sports and increased numbers of participating countries and athletes. In light of this, one could speculate that the race time differences between the skiers have gotten smaller also in Para XC skiing, but this has not yet been examined.

Therefore, the primary aim of this study was to compare differences in race time (RT_diffs_) between female and male Para and AB XC skiers. To investigate the effects of the time-factor on RT_diffs_ for the Para skiers, analyses were done both with the adjusted and unadjusted race times. The secondary aim was to examine how RT_diffs_ changed across the 2011–2020 seasons. For the primary aim, we hypothesized to find: (1) larger RT_diffs_ among Para compared to AB skiers, (2) larger RT_diffs_ among female skiers compared to male skiers, and (3) smaller RT_diffs_ with adjusted compared to the unadjusted race times. For the secondary aim, we hypothesized that RT_diffs_ for the Para skiers were reduced from the 2011 to the 2020 season.

## Materials and Methods

### Overall Design

Race data of Para and AB skiers during all WCs, WCHs, and PWG/OWGs were analyzed for the 2011–2020 seasons. The data were limited to the 2011–2020 seasons, as competition results for Para skiers have been systematically stored by the IPC since the 2010/2011 season. Lower-level skiers may disproportionally influence RT_diffs_, and lead to larger differences across the competition field compared to those seen among the medalists. Accordingly, RT_diffs_ for each individual race were calculated for both the top-8 and the top-3 skiers for all skiing groups (VI, STAND, SIT, and AB skiers).

### Data Extraction

Official race data of Para and AB skiers was extracted from the IPC (IPC, 2021b) and FIS websites (FIS, 2021), respectively. The IPC and FIS obtain informed consent from all competing athletes to publish the race data online. Prior to any processing, we de-identified the data through the removal of identifiable information. The data extraction and processing were approved by the Norwegian Centre for Research Data (ID 765557).

For every time-trial competition, sex, race time, final rank, race distance, skiing style, event type, and season were extracted for each Para and AB skier. Additionally, information on the skiing category and class were extracted for the Para skiers. To obtain the unadjusted race times, each adjusted race time was divided by the class-specific time-factor of the 2019/2020 season. The time-factor is evaluated every season by the IPC and while minor changes may have been made during the 2011 to 2020 seasons, these are unlikely to have a major effect on our calculations. Only data from individual short- and middle-distance time-trial races were included in the analysis. Data of the sprint- and long-distance races were excluded since these are mass start competitions for AB skiers, and are known to affect the pacing strategy and speed, and thereby race times (Thiel et al., 2012; Hanley, 2015; Losnegard, 2019). The division of short- and middle distances was done as per IPC (IPC, 2020) and FIS (FIS, 2020) regulations (Table 1). It should be noted that the SIT skiers only compete in the classical technique so their data is limited to this skiing style.

**Table 1:** The number of races included in the analyses divided for Para (SIT: physically impaired sitting skiers, STAND: physically impaired standing skiers, and VI: visually impaired standing skiers) and AB skiers by sex, skiing style, and distance.

	**Skiers**	**Races (No.)**	**Sex**	**Races (No.)**	**Skiing style**	**Races (No.)**	**Distance**	**Length (km)**	**Races (No.)**
Para	SIT	61	M	37	Classical	37	Short	5	8
							Middle	10	29
			F	24	Classical	24	Short	5	5
							Middle	7.5	19
	STAND	46	M	25	Classical	17	Short	7.5	4
							Middle	12.5	13
					Free-style	8	Short	7.5	2
							Middle	12.5	6
			F	21	Classical	10	Short	5	2
							Middle	10	8
					Free-style	11	Short	5	3
							Middle	10	8
	VI	24	M	18	Classical	9	Short	7.5	2
							Middle	12.5	7
					Free-style	9	Short	7.5	1
							Middle	12.5	8
			F	6	Classical	4	Short	5	2
							Middle	10	2
					Free-style	2	Short	5	–
							Middle	10	2
	AB	104	M	49	Classical	23	Short	10	4
							Middle	15	19
					Free-style	26	Short	10	8
							Middle	15	18
			F	55	Classical	17	Short	5	3
							Middle	10/15	14
					Free-style	38	Short	5	9
							Middle	10/15	29

*F, Female skiers; M, Male skiers*.

### Data Processing

Races with fewer than 10 competitors were excluded (58, 39, 29, and 1% for VI, STAND, SIT, and AB skiers, respectively) to reduce inflation of RT_diffs_ due to lower-level skiers. For the same reason, only results of the top-8 skiers were included and the results of the skiers ranked 9^th^ and 10^th^ place were excluded. The percent RT_diff_ between the winner and the other seven skiers (i.e., 2nd to 8th rank) were calculated for all remaining races. For the Para skiers, this was done for both the adjusted and unadjusted race times.

### Statistical Analysis

All statistical analyses were performed using linear mixed modeling procedures in Stata 16.1 (StataCorp LLC, College Station, Texas, USA). For the primary aim, we investigated the main effect of skiing group (i.e., VI, STAND, SIT, and AB skiers) and sex, as well as the interaction between skiing group^*^sex on RT_diffs_ for the top-3 and top-8 skiers. In the latter analyses, only the adjusted race times were used for the Para skiers. Separate analyses were performed to investigate whether RT_diffs_ differed between Para XC skiing groups when using the unadjusted or adjusted race times. For the secondary aim, we investigated the main effect of season and skiing group, as well as the interaction between the season^*^skiing group on RT_diffs_ for the top-3 and top-8 skiers. For these analyses, the adjusted race times were used for the Para skiers. A separate analysis was performed for the Para XC skiing groups to investigate whether the RT_diffs_ between seasons were larger when using the unadjusted race times compared to the adjusted race times. All the models were adjusted for the fixed factors distance (short or middle), skiing style (classical or free-style), and event type (WC, WCH, or PWG/OWG). *Post-hoc* analyses using the Bonferroni's correction were performed for pairwise comparisons of the estimated marginal means for the skiing group, skiing group^*^sex, and skiing group^*^season. Visual examination of Q–Q plots, and plots comparing residual vs. predicted values indicated no deviation from normality. Results are reported as mean ± 95% CI if not stated otherwise. An alpha-value of 0.05 was used to indicate statistical significance.

## Results

### Differences in Race Time Across Skiing Groups and Sex

**Figure 1:**
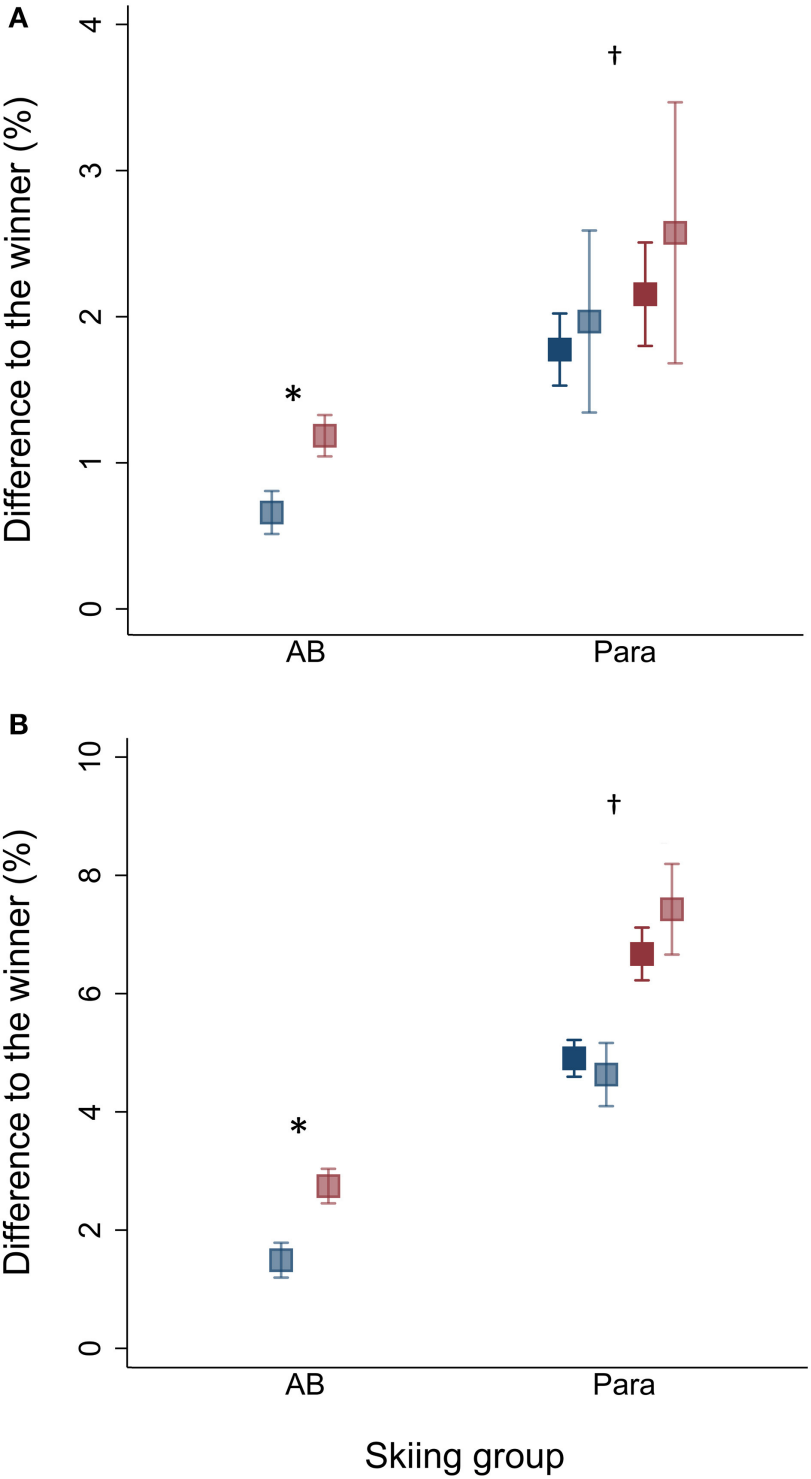
Estimated adjusted (dark color) and unadjusted (light color) differences in race time (RT_diffs_) for **(A)** top-3 and **(B)** top-8 female (red) and male (blue) Para and able-bodied (AB) skiers. Presented as mean ± 95% CI. *Significantly larger adjusted RT_diffs_ for female compared to male skiers within same skiing group, *p* < 0.05. Significantly different from AB skiers, *p* < 0.01.

**Figure 2:**
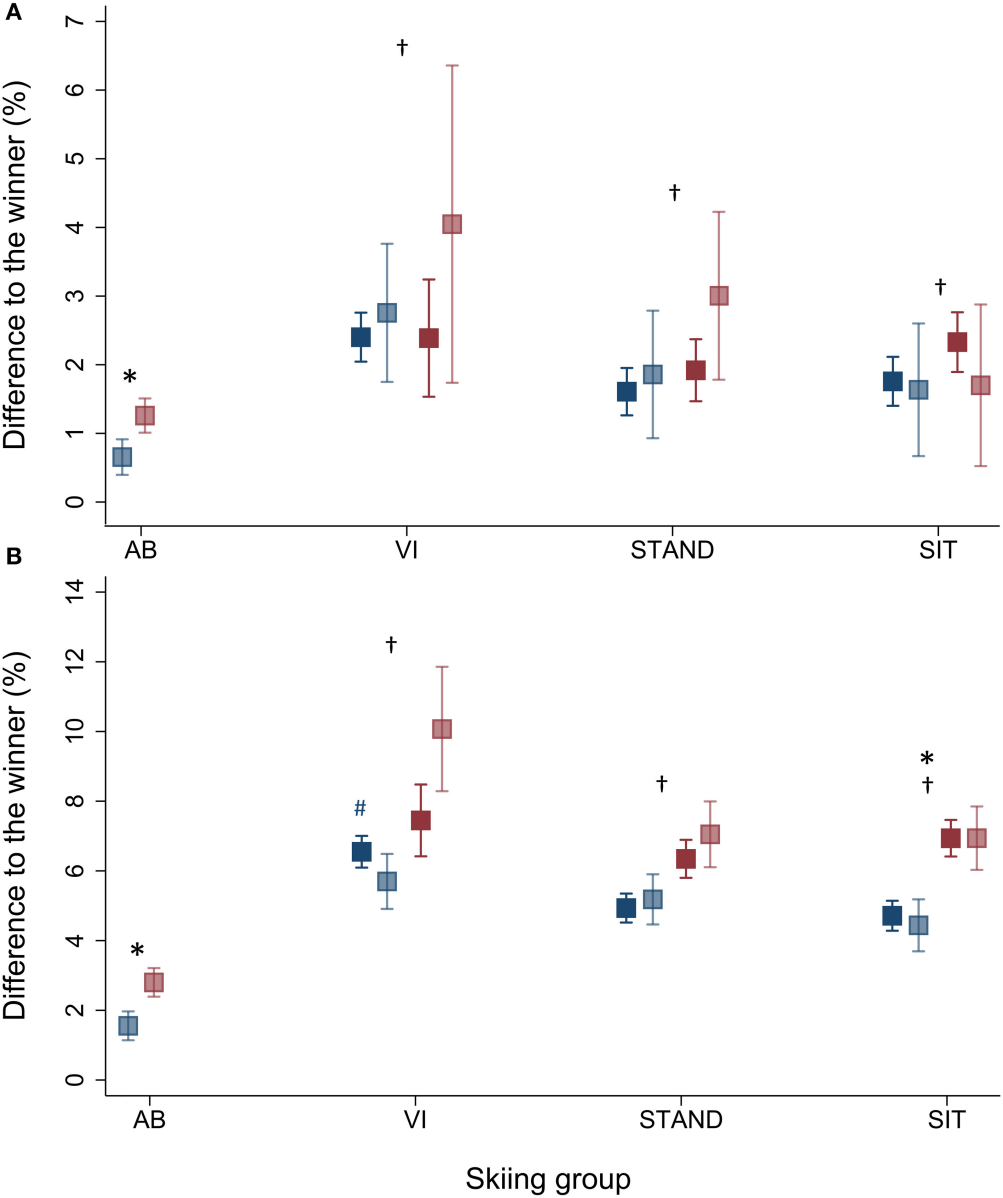
Estimated adjusted (dark color) and unadjusted (light color) differences in race time (RT_diffs_) for **(A)** top-3 and **(B)** top-8 female (red) and male (blue) Para (VI: visually impaired standing skiers, STAND: physically impaired standing skiers, and SIT: physically impaired sitting skiers) and able-bodied (AB) skiers. Presented as mean ± 95% CI. *Significantly larger RT_diffs_ for female compared to male skiers within same skiing group, *p* < 0.05. Significantly different from AB skiers, *p* < 0.01. ^#^Significantly larger adjusted RT_diffs_ for male VI compared to male STAND and SIT skiers, *p* < 0.05.

When adjusted for sex, RT_diffs_ were significantly larger in Para compared to AB for both the top-3 and top-8 skiers (top-3: 2.1 vs. 0.9%; top-8: 6.2 vs. 2.1%, all *p* < 0.01) (Figure 1). There were no significant differences in RT_diffs_ among the top-3 skiers across the Para categories (all *p* > 0.2). However, RT_diffs_ among the top-8 skiers were significantly larger for VI compared to STAND and SIT (7.0, 5.5, and 5.6%, respectively; all *p* < 0.05) (Figure 2).

Furthermore, when adjusted for skiing groups, RT_diffs_ were significantly larger among female compared to male skiers (top-3: 1.8 vs. 1.3%; top-8: 4.9 vs. 3.5%, all *p* < 0.05) (Figure 2). RT_diffs_ among top-3 skiers were significantly larger for female compared to male AB skiers (1.3 vs. 0.6%, *p* < 0.02), while there were no significant differences between female and male Para skiers within any of the Para categories (all *p* > 0.1). RT_diffs_ amongst the top-8 skiers were significantly larger for female compared to male SIT (7.0 vs. 4.5%, *p* < 0.001) and AB (2.8 vs. 1.6%, *p* < 0.001).

There were no significant differences between adjusted and unadjusted RT_diffs_ for neither the top-3 nor top-8 skiers in any of the Para categories (all *p* > 0.1) (Figure 2).

### Differences in Race Time Across Seasons

**Figure 3:**
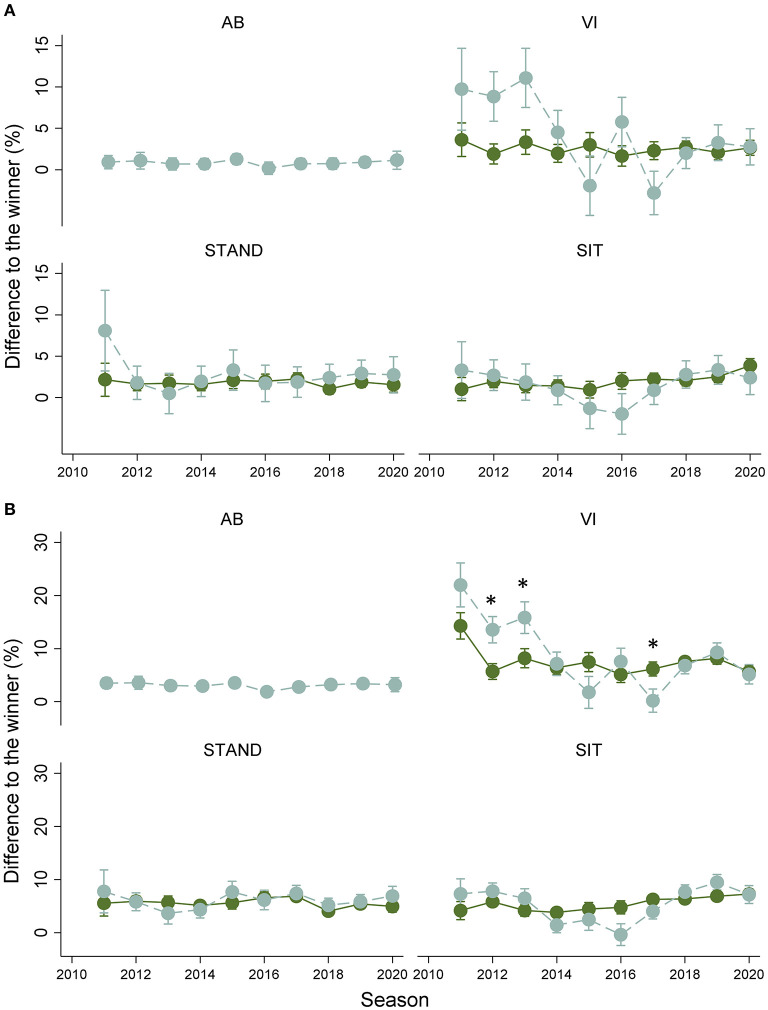
Estimated differences in race time (RT_diffs_) for **(A)** top-3 and **(B)** top-8 of adjusted (dark green solid line) and unadjusted (light green dashed line) race times of Para (VI: visually impaired standing skiers, STAND: physically impaired standing skiers, and SIT: physically impaired sitting skiers), and able-bodied (AB) skiers for the 2011–2020 seasons. Values presented as an annual average ± 95% CI. *Significant difference between adjusted and unadjusted RT_diffs_, *p* < 0.05.

When adjusted for sex and skiing group, RT_diffs_ for the top-3 and top-8 skiers did not significantly differ across the seasons 2011–2020 for the top-3 skiers (VI: 3.6–2.6%, STAND: 2.2–1.6%, SIT: 1.0–3.9%, AB: 1.0–1.1%, all *p* > 0.1) and top-8 skiers (VI: 12.0–4.2%, STAND: 2.6–3.5%, SIT: 1.9–5.9%, AB: 1.5–0.9%, all *p* > 0.1). As such, the comparisons of RT_diffs_ between skiing groups and sex provided in the previous section are similar across seasons. Furthermore, there was no significant difference between adjusted and unadjusted RT_diffs_ for the top-3 and top-8 skiers across the seasons 2011–2020 (all *p* > 0.1) (Figure 3).

## Discussion

This study investigated RT_diffs_ for the top-3 and top-8 female and male Para and AB skiers. Para skiers displayed larger RT_diffs_ than AB skiers, and female skiers displayed larger RT_diffs_ than male skiers. There were no significant differences between RT_diffs_ when using the adjusted or unadjusted race times, but the variability of RT_diffs_ within the Para categories was slightly reduced after the time-factor adjustments. RT_diffs_ were stable across the last 10 seasons, but displayed a slightly less variable pattern for adjusted race times compared to unadjusted race times.

### Differences in Race Time Across the Skiing Groups

This is the first study to examine RT_diffs_ between Para and AB skiers, as well as between the three Para categories. In support of our primary hypothesis, Para displayed larger RT_diffs_ compared to AB skiers, which also supports the earlier study on elite Para and AB swimmers (Fulton et al., 2009). The authors related these findings to the effects of disability on performance, more limited international race experience, and a slower evolution of the sport (Daly and Vanlandewijck, 1999; Fulton et al., 2009), which may also explain the larger RT_diffs_ in our study. Additionally, the lower number of competitors in Para XC skiing means that a larger proportion will be included in the top-8. The range of performance levels in the top-8 is therefore likely larger compared to AB skiers, which contributes to larger RT_diffs_. This is supported by our observation that the Para skiers had a ~4 percentage points increase of RT_diffs_ from the top-3 skiers to top-8 skiers (from 2.1 to 6.2%), while the AB skiers only had a ~1 percentage point increase (from 0.9 to 2.1%).

Notably, VI displayed significantly larger RT_diffs_ compared to SIT and STAND for the top-8 skiers, which likely can be attributed to differences between the male competitors (Figure 2B). Larger disability-related performance differences between the male Para XC skiing categories may be due to a larger proportion of the male VI competitors in the lower classes, which include skiers with the largest functional limitations. In addition, there is a possibility that the general competitiveness (i.e., not related to the disability) may be lower in male VI skiers compared to SIT and STAND competitors, however, the reasons for such differences are currently unclear.

In contrast to our hypothesis, RT_diffs_ for the Para skiers have remained relatively stable across the last 10 seasons. This is similar to previous findings in AB XC skiing (Stöggl et al., 2009; Spencer et al., 2014), but differs from the observation of closer race times in Para sprint running between 1992 and 2012 (Grobler et al., 2015). Two lines of reasoning could explain the stable RT_diffs_ across seasons among the Para skiers. Either a performance development has not happened during the examined time period (i.e., 2011–2020), or the performance development was there but similar for all levels of the Para skiers, leaving RT_diffs_ unchanged.

### Differences in Race Time Between Female and Male Skiers

In support of our hypothesis, RT_diffs_ were larger in female skiers compared to male skiers. Notably, this difference was significant only for the AB skiers, while there were no significant differences between the Para skiers which are likely attributed to the large variability and small sample sizes within the female Para skiers. Larger RT_diffs_ in female athletes have previously been demonstrated in AB XC skiing (Stöggl et al., 2009; Spencer et al., 2014), skeleton (Bullock et al., 2009), and slalom kayaking (Nibali et al., 2011), and explained by lower performance levels of the best female compared to the best male athletes (Bullock et al., 2009; Spencer et al., 2014). The same seems to be the case for female Para skiers, especially given that female races typically have half the number of competing skiers than male races do (i.e., 24 female Para skiers vs. 42 male Para skiers on average in each category).

### Adjusted and Unadjusted Differences in Race Times

For the top-3 and top-8 skiers, neither VI, STAND, nor SIT skiers displayed a significant difference between adjusted and unadjusted RT_diffs_. This is contrary to our hypothesis and likely attributed to the large variability in RT_diffs_, especially for the unadjusted RT_diffs_. While not being significantly different, unadjusted times still displayed a more variable pattern across the last ten seasons compared to the corresponding adjusted times. Accordingly, the time factor appears to contribute to less variability in RT_diffs_ among the Para skiers, especially for VI (Figures 3A,B). Speculatively, the more variable patterns in RT_diffs_ based on the unadjusted race times for VI may be related to a larger proportion of skiers in lower functional classes. Future investigations should look into the differences between adjusted and unadjusted RT_diffs_ not only within Para XC skiing categories, but also within classes.

### Methodological Considerations

In the current study, the calculations for RT_diffs_ differed slightly from other studies investigating differences in race time [e.g., AB XC skiing (Spencer et al., 2014) and Para swimming (Fulton et al., 2009)]. In these studies, race times were log-transformed prior to analyses, which yield the variability and differences as percentages of the mean when back-transformed. In this study, the differences in race times as percentages were directly calculated from the absolute race times before the analyses. Therefore, caution is warranted when directly comparing our results with findings from other studies. In addition, in the current study, the time-factor system in place at the 2019/2020 seasons was employed for all ten seasons. While the time-factor is evaluated every season by the IPC (IPC, 2016), and minor changes may have been made during the 2011–2020 seasons, these are unlikely to affect the general outcome of the comparisons between adjusted and unadjusted RT_diffs_.

## Conclusion

Para skiers displayed larger RT_diffs_ compared to the AB skiers. This is likely due to disability-related differences in performance among the Para skiers and fewer competing skiers compared to the AB equivalents. The larger RT_diffs_ for female skiers compared to male skiers were predominantly due to differences within the AB skiers, and lower performance levels in AB females. While differences were also larger in female compared to male Para skiers, this did not reach statistical significance, likely due to variability and small sample sizes within the female Para skiers. Using adjusted or unadjusted race times did not affect RT_diffs_ significantly, although the adjusted times were less variable and thus indicating that the time factor contributes to lower RT_diffs_ between the Para skiers. These findings are important for athletes and coaches during the goal-setting process and for evaluating the training progress, as they indicate the performance improvement needed to gain a better rank or win the race. In future studies, the distribution of classes within the categories, and its relationship to the differences in race time, should be investigated.

## Data Availability Statement

The original contributions presented in the study are included in the article/supplementary materials, further inquiries can be directed to the corresponding author.

## Ethics Statement

The studies involving human participants were reviewed and approved by the Norwegian Centre for Research Data. Written informed consent for participation was not required for this study in accordance with the national legislation and the institutional requirements.

## Author Contributions

CHC, JKB, and ØS all contributed to the conceptualization and design of the study. CHC and JKB acquired the data. CHC, JKB, and CS interpreted the results. CHC analyzed the data and drafted the study with all authors critically revising it for important intellectual content. All authors approved the final version to be published.

## Funding

This study was funded by the Centre for Elite Sports Research, Department of Neuromedicine and Movement Science, Norwegian University of Science and Technology, Trondheim, Norway.

## Conflict of Interest

The authors declare that the research was conducted in the absence of any commercial or financial relationships that could be construed as a potential conflict of interest.

## Publisher's Note

All claims expressed in this article are solely those of the authors and do not necessarily represent those of their affiliated organizations, or those of the publisher, the editors and the reviewers. Any product that may be evaluated in this article, or claim that may be made by its manufacturer, is not guaranteed or endorsed by the publisher.
